# Measuring dementia carers' unmet need for services - an exploratory mixed method study

**DOI:** 10.1186/1472-6963-10-122

**Published:** 2010-05-13

**Authors:** Christine Stirling, Sharon Andrews, Toby Croft, James Vickers, Paul Turner, Andrew Robinson

**Affiliations:** 1 Wicking Dementia Research and Education Centre, Menzies Research Institute, University of Tasmania, Private Bag 121, Hobart TAS, 7000 Australia; 2 Department of Psychology, Royal Hobart Hospital, Liverpool St, Tasmania, Australia; 3 School of Medicine, University of Tasmania, Collins St, Australia; 4 School of Computing and Information Systems, University of Tasmania, Churchill Ave, Australia; 5 School of Nursing and Midwifery, University of Tasmania, Campbell St, Australia

## Abstract

**Background:**

To ensure carers of people with dementia receive support, community services increasingly use measures of caregiver (carer) burden to assess for unmet need. This study used Bradshaw's taxonomy of need to explore the link between measures of carer burden (normative need), service use (expressed need), and carer's stated need (felt need).

**Methods:**

This mixed method exploratory study compared measures of carer burden with community services received and unmet needs, for 20 community-dwelling carer/care-recipient pairs.

**Results:**

A simple one-item measure of carers' felt need for more services was significantly related to carer stress as measured on the GHQ-30. Qualitative data showed that there are many potential stressors for carers, other than those related to the care-giving role. We found a statistically significant rank correlation (p = 0.01) between carer's use of in-home respite and the care-recipient's cognitive and functional status which is likely to have been related to increased requirement for carer vigilance, effort and the isolation of spouse carers. Otherwise, there were no statistically significant relationships between carer burden or stress and level of service provision.

**Conclusion:**

When carers are stressed or depressed, they can recognise that they would like more help from services, even if measures of carer burden and care recipient status do not clearly indicate unmet service needs. A question designed to elicit carer' *felt *need may be a better indicator of service need, and a red flag for recognising growing stress in carers of people with dementia. Assessment of service needs should recognise the fallibility of carer burden measures, given that carer stress may not only come from caring for someone with dementia, but can be significantly compounded by other life situations.

## Background

Assessment and monitoring of caregiver (carer) burden are increasingly seen as essential factors in ensuring that carers receive community support [1], but the outcomes of this approach are uncertain. While meeting carer service needs has been the subject of increased policy and research interest, Bradshaw's [2] taxonomy of need has not been utilised in this context. Intervention studies often show non significant findings, and researchers increasingly question the usefulness of existing measures of carer burden [3,4]. It follows then that using these same measures to assess 'carer's service needs may generate misleading outcomes. This study contributes to knowledge about the options for assessing carer's unmet needs [4-6]. We argue here, that the complexity of carer's needs make it difficult to rely on measures of carer burden and care-recipient dependency for assessing carer's needs.

The focus of this study is carers of persons with dementia, who provide critical support for care recipients by improving their quality of life and delaying entry to care homes [7]. With estimates of 63 million people with dementia globally in 2010 [8] and in recognition of the important economic and quality of life benefits provided by carers, policy makers are urgently seeking ways to ensure the ongoing capacity of carers to provide the bulk of care. 'Carer burden' is a term used to describe the negative effects of caring on carers' physical, mental, social, and financial well-being. Such effects are clearly evident in the context of caring for people with dementia [9,10]. Factors such as carer resources, the tasks of care (such as managing the behavioural and psychosocial symptoms of people with dementia), carer attributes, and the relationship between carers and carer recipients, interact in complex ways with carers' coping and wellbeing [1,5,6,11-13]. Service providers have attempted to capture this complexity through an increasing array of carer and care-recipient assessments, striving to prevent carer burnout by meeting carer' service needs [14].

In comparison with the general carer population, carers of people with dementia exhibit higher levels of unmet need and lower levels of service use [3,15,16]. This is problematic because the resultant physical and emotional distress for carers [17,18], is strongly predictive of impending entry to care homes or the death of the care recipient [18,19]. Community services are commonly seen as a key intervention for reducing carer burden, despite the inconsistent nature of research evidence [18,20]. Services such as respite, nursing assistance, domestic assistance, and personal care aim to support carers and people with dementia, so that ageing in place can be maintained as long as possible. Low levels of service use however, suggest that there are unrecognised obstacles to assessing and meeting carer needs [14]. Respite care, for example, is one of the major forms of assistance directly targeted to carers [21], but in Australia this service is not used by up to 70% of carers [22].

Brodaty et al. [23] developed a typology of four reasons for service non-use in dementia carers: 'managing at the moment', 'reluctant to use services', 'service characteristics', and 'do not know about services'. Studies show that a complex range of socio-cultural factors is implicated in carer 'reluctance to use services', including carer identity barriers, fear of role change, concerns for privacy, financial factors, and personal characteristics [11,13,23]. This complex causality of carers' service needs has to date been addressed through an increasing reliance on comprehensive assessments of carer need.

Service organisations typically use a range of assessment tools at admission to assess carers' service needs, which generally act as a 'gateway' to services. In this approach, a care professional "begins with the identification of specific difficulties, accounts for the presence and efficacy of current help, recognises perceived need and finally specifie[s] the type of intervention required to meet those needs" [[3]: 323]. Within this paradigm, the care professional focuses assessment on the status of the person with dementia, the subsequent perceived burden on the carer, and any existing socio-economic support deficits [3]. However, given the increasing awareness of the complexity of factors that may be responsible for unmet need [18], such an approach promises an increasing cost in terms of time and other resources.

Given the recognised socio-cultural complexity of carers unmet needs, alternatives to the professional assessment paradigm is needed [24]. We adopted Bradshaw's [2] sociological typology of need as one that might best capture the complexity of carers' situations. Categories of need defined by Bradshaw [2] are *normative *need, *felt *need, *expressed *need, and *comparative *need. The carer burden assessment approach described by Meaney et al. [3] is a *normative *view of need, which is professionally identified. The term *felt *need identifies needs articulated by potential service users themselves. This need is captured by the simple process of asking people what services they would like. *Expressed *need is the actual demand or uptake for services. The low rate of translation of *felt *need into *expressed *need in carers' orientation towards services can be explained by a range of socio-cultural factors. Finally, *comparative *need is assessed by comparing groups of service users with groups of non-service users to compare characteristics. No single form of measure is likely to capture all carers' unmet needs, but the move towards person-centred care has increasingly challenged *normative *need as paternalistic and inappropriate [3].

This study aimed to explore the relationship between different types of carer service need using Bradshaw's [2] typology. The in-depth study used a mixed method design with a concurrent triangulation strategy [25] to investigate a group of carers and care recipients with dementia. Quantitative subjective and objective measures of carer burden and carer stress were administered and community service use measured, thereby capturing indicators of *normative, felt, and expressed need*. Qualitative in-depth interviews were conducted at three time points, in order to assess the context of service use and carer need, and to capture the complexity of socio-cultural contexts.

## Methods

### Participants

The study population consisted of 20 community-dwelling pairs of dementia carers and people with dementia. Carers were required to identify as a 'primary carer' - that is, the person providing the greatest amount of care - and could be either co-habiting with a person with dementia or living apart. Carers (n = 24) known to the local Alzheimer's Australia organisation were contacted and invited to participate, with 20 consenting. This method of recruiting ensured that carers were caring for someone with a dementia diagnosis, and that they were linked into some formal support, irrespective of their concurrent use of other community services. Isolated carers without any links to community services were not represented in the sample. Ethics approval was received for this study from the Tasmanian Social Science Human Research Ethics Committee.

### Procedures

Four visits took place with each carer over 12 weeks, at weeks 1, 4, 8 and 12. During the first visit, as data collection was occurring with carers, care recipients were seen by a psychologist in a separate location in their homes. Carers completed self-report measures on carer burden and stress (*normative *need measures), indicated their service wants (*felt *need measures), kept a service usage diary over the 12 week study period (*expressed *need measures), and participated in three semi-structured interviews, conducted at monthly intervals. Data was compared across participants.

### **Qualitative data**

The interviews lasted between 30 to 90 minutes and elicited data on the nature, frequency and quality of carers' interactions with community service providers. Carers' experiences of their carer role were also explored, in order to provide a context for their interactions with service providers. Carers were engaged in a process of progressive disclosure during the interviews, in which semi-structured research questions guided them through a series of increasingly sensitive topics. For example, in interview 2, carers were asked to clarify the services they had been using (and had documented in the service usage diary) and to expand on their interactions with service providers. In later interviews (3 and 4), questions centred on the carers' socio-economic circumstances, their *felt *need for more or different services and their care-giving experience.

### Measures of Care Recipient Dependency

Carer burden (*normative *need) was assessed by measuring the severity of dementia and functional dependency of the care recipient, as well as carers' subjective ratings of burden. Two tools were used to obtain an indication of the severity of dementia in the care recipient. The Dementia Rating Scale-2 (DSR-2) was administered, a tool widely used to screen cognition in people with a known or suspected dementia. This tool provides objective, psychometric measures of attention, construction, initiation/perseveration, conceptualisation and memory, with lower scores indicating higher deficits [26]. Carers also completed the Bayer Activities of Daily Living Scale (BADLS) [27]. This informant-rated questionnaire assesses functional disabilities across 25 everyday tasks, using a ten-point Likert scale to assess difficulty: (1 = never has difficulty, 10 = always has difficulty), with the additional options of "I don't know' and 'Not applicable'. Higher scores correspond to higher deficits.

### Measures of Carer Burden

Indicators of carer burden and mental health status were obtained at time point 1 (*normative *need). A researcher-administered Carers' Checklist [28] captured carers' perceptions of burden in domains relevant to care recipients and carers' functioning [29]. The Carers' Checklist addresses a number of domains relevant to the functioning of the person with dementia and his or her carer. For the person with dementia, this includes: cognitive and psychological symptoms, ADLs and self care, inappropriate behaviours, social behaviours and safety issues. To assess the extent of behavioural and psychological problems in the care recipient, carers indicate whether any problem behaviours are exhibited by the person with dementia: ("always", "sometimes", "never"). The Carers' Checklist also includes items on the care-recipients' assistance needs for Activities of Daily Living (ADLs) and self care, social behaviours and safety issues. 'Objective' burden is indicated by means of carers' reports as to how often these 26 dementia-related problems occur (BPSDs, ADLs etc.), while 'subjective' burden is measured by how stressful carers rate each of these problems [28]. Carer perceptions of overall burden are obtained through five scales, which ask carers about physical, financial, emotional and social burden. Carers rate how burdensome they find caring on each scale from 1 (no burden at all) to 5 (a great burden) [28]. The Carers' Checklist has been used across a range of service settings, including the community [29], and has shown high internal consistency (Cronbach's alpha = 0.93) [28].

The General Health Questionnaire-30 item version (GHQ-30) was also administered at time point 1. This is designed as a first stage screening tool for psychiatric illness, to provide an objective indicator of non-psychotic psychiatric disorders (typically anxiety and mood disorders) [30]. A GHQ score of 5 or above indicates an increased likelihood of a non-psychotic mental health problem which would warrant subsequent treatment measures [30].

### Service Usage

The final domain covered by the Carers' Checklist is carers' *felt *need for services. This is captured through four items that ask carers whether they need more help from services than is given, want better access to services, want more information than is given, or feel that services should work together and communicate more effectively. Carers responded using a 3 point scale: (never, sometimes and always).

Service usage (*expressed *need) was captured through service diaries. Carers reported the weekly hours of community services received, with new diaries provided each month. Weekly support phone calls and monthly face to face assistance at the time of the interviews formed the cornerstone of a progressive engagement approach, which was aimed at maintaining carers' engagement with the project and facilitating complete and accurate data collection. At the final interview, a question about the carers experience of participating in the study, aimed to bring a natural, seamless, and positive closure for them. Disengagement was also assisted by presenting a (unexpected) gift voucher in appreciation of participants' contribution.

### Analysis

Bivariate descriptive analysis with categorical data used Kendall's tau-*b *for ordinal data, with square tables and Spearmans rho for variables with more categories. Service variables from the service usage diaries were categorised into: (i) Practical assistance, encompassing domestic help, gardening help and physical care; (ii) in-home respite, incorporating any service delivered in the family home, primarily to provide diversional activities or supervision for the person with dementia; (iii) out-of-home respite, incorporating any service that was situated in the community, designed for people with dementia, and relieved carers of the caring role, and; (iv) a new dichotomous variable that was created for each service type of any or no service use.

All interview data were transcribed and subjected to case analysis by two members of the research team. Discussions about services and usage of services were highlighted. Cases that exhibited *normative *need (i.e had low service usage according to burden measures in comparison with other cases) were investigated, in order to uncover possible explanations. Members of the research team engaged in peer debriefing, exploring rival explanations, probing biases, and clarifying the basis of interpretation, with a view to enhancing the credibility of the analysis [31].

## Results

### Participant demographics

Table 1 summarises the demographic data and Table 2 the clinical characteristics of the sample. Our sample of carers had characteristics consistent with the demographic profile of carers of people with dementia in Australia, although males were slightly under-represented [22]. Most were female (90%), co-resident carers (85%), the spouse of the care recipient (70%), and aged over 66 years. Our care-recipient sample was consistent with known characteristics of this group, in terms of age, and dementia cause [22]. The majority of care recipients had a formal diagnosis, with 55% having Alzheimer's disease, 15% vascular dementia, 20% dementia of an unknown/unspecified cause and 5% Parkinson's disease or fronto-temporal dementia. Despite the relatively small sample size, participants' characteristics fitted within the range of those reported in studies of Australian carers and people with dementia, though we had a higher proportion of female carers and more male care-recipients. Limited data about carers of people with dementia has been generated within Australian contexts. Table 1 includes a column where available national statistics from AIHW (2007) are included.

**Table 1:** Demographic characteristics of 20 caregivers and 20 care recipient participants.

Participant Characteristics		**N %**,
***Carer Characteristics***	National Characteristics *	
**Carer, Male/Female**	female < 70%	2 (10)/18 (90)
**Carer Age**	95% > 60 yr	
65 yr or less		5(25)
66 to 75 yr		10(50)
76 yr or older		5(25)
**Carer Highest Education Level**		
School or Certificate	73-76%	16(80)
Degree or Higher		4(20)
**Live with Care Recipient**, Yes/No	(65)/(35)	17 (85)/3 (15)
**Years of Caring**		
< 1 year		1 (5)
1 - 2 years		4 (20)
2 - 3 years		9 (45)
> 3 years		6 (30)
**Care Relationship**		
Spouse	(65%)	14 (70)
Relative	(30%)	5 (25)
Other		1 (5)
**Previous Care Experience**, Yes/No		8 (40)/12 (60)
***Care Recipient Characteristics***		
Care Recipient Age 75 or less/>75	**(27%) /(73%)	7 (35)/13 (65)
Care Recipient, Male/Female	***(52)/(48)	15 (75)/5 (25)

*Taken from AIHW [22]

**Calculated from Table 4.3 AIHW [22] using 'household' figures

*** In cases where the primary carer is a co-resident

**Table 2:** Objective and subjective clinical characteristics of the 20 caregiver and 20 care recipient participants.

Subjective and Objective measures of burden	Statistics mean (range) ± SD
DRS2, mean [*CI *α = 0.05]	95 [*83-107*] (35-130) ± 25
BADLS Total, mean [*CI *α = 0.05]	7.6 [*7-8*] (4-10) ± 1.8
Care Recipient Age at Diagnosis,	74 (53-90) ± 9
Formal Diagnosis, Yes/No	*N (%) 18 (90)/2 (10)*
**Measures of Carer Burden**	
GHQ-30, mean [*CI *α = 0.05] *scoring 5 or more*	8 [*5-12*] (0-27) ± 7 *N (%) 13 (65)*
CCL-CR Behaviour where max possible = 52	19 (8-37) ± 8
CCL-Carer stress where max possible = 52	15 (3-32) ± 8
CCL-Overall burden	3 (2-5) ± 1
CCL-Physical burden	3 (1-5) ± 1
CCL-Emotional burden	4 (1-5) ± 1
CCL-Financial burden	2 (1-5) ± 1
CCL-Social burden	3 (1-5) ± 1
CCL-Carer rated stress by service needs	2 (0-8) ± 3
**Services Provided**	
Total out of home respite hours over 3 months *receiving ANY hrs*	52 (0-294) ± 72 *N (%) 14 (70)*
Total in home respite over 3 months *receiving ANY hrs*	29 (0-119) ± 34 *N (%) 11 (55)*
Total practical help over 3 months *receiving ANY hrs*	22 (0-125) ± 32 *N (%) 12 (60)*
**CCL-carer feels need more from services**	3 (0-8) ± 2
Need more help from services than I am given - yes,	*N (%) 13 (65)*
Need more information than I am given - yes,	*N (%) 8 (40)*
Services should work together & communicate more - yes	*N (%) 10 (50)*
Need better access to services - yes	*N (%) 11 (55)*

### Care Burden - normative need

The DRS-2, BADLS and Carers' Checklist scores indicate that care recipients were moderately to severely impaired with dementia. Seventy percent scored at or below the 1^st ^percentile on the total DRS-2 score, indicating pronounced cognitive impairment significantly affecting a wide range of everyday activities. The most commonly reported behaviours were forgetfulness (11/20 always, and 8/20 sometimes) and always asking questions (9/20 always and 6/20 sometimes). Additionally, eleven care recipients "could not be left alone even for one hour" and eight "wandered at night". The total BADLS scores had a positive relationship (Spearmans rho = 0.649, p = 0.01) with the carer reported dementia related problems, as could be expected, but there was no relationship between mean behaviour scores and GHQ-30 scores.

Results for measures of carer stress and strain were obtained through the Carers' Checklist and the GHQ 30, and are presented in Table 2. Carers self-rated a 'moderate strain' (mean of 3 out of 5) overall, and in the physical and social domains of caring. Emotional strain was rated at the point halfway between 'moderate strain' and 'a great strain', whilst financially, carers indicated a lower strain. The mean GHQ-30 score for carers was nearly double that of their non care-giver peers (8 versus 4.72) [30] with two-thirds (compared to 33% of their peers) scoring 5 or over. These results indicate significantly elevated levels of psychiatric symptoms (likely to be predominantly anxiety and depression) in this sample, despite carers' subjective assessments of more moderate burden. We found no significant correlation between measures of dementia severity and the GHQ-30 scores.

Overall, the mean carer self-rating for stress linked to dementia behaviours was 15, out of a total possible score of 52. At time point 1, care recipient behaviours most commonly rated as 'very stressful' or quite stressful' by carers were 'forgets things which have happened' (7/20 - very stressful; 8/20 - quite stressful), 'always asking questions' (7/20 - very stressful; 7/20 quite stressful), 'not safe to be in the house alone' (5/20 - very stressful; 7/20 - quite stressful); 'cannot be left alone even for one hour' (5/20 - very stressful; 5/20 - quite stressful) and 'wanders at night' (4/20 - very stressful; 1/20 - quite stressful). These measures demonstrated that within our sample. carers exhibited high levels of *normative *need on objective carer burden measures, and moderate levels of *normative *need on subjective carer burden measures.

### Service Usage - expressed need

Service usage summary data is detailed in Table 2. The most used service was out-of-home respite, which had a greater range of hours because the category included overnight respite. Most carers received a combination of services, with 25% (n = 4) of the sample receiving all three types of services, 30% (n = 6) received both out-of-home respite and practical assistance, and 15% (n = 3) used both in-home respite and out-of-home respite services.

**Figure 1:**
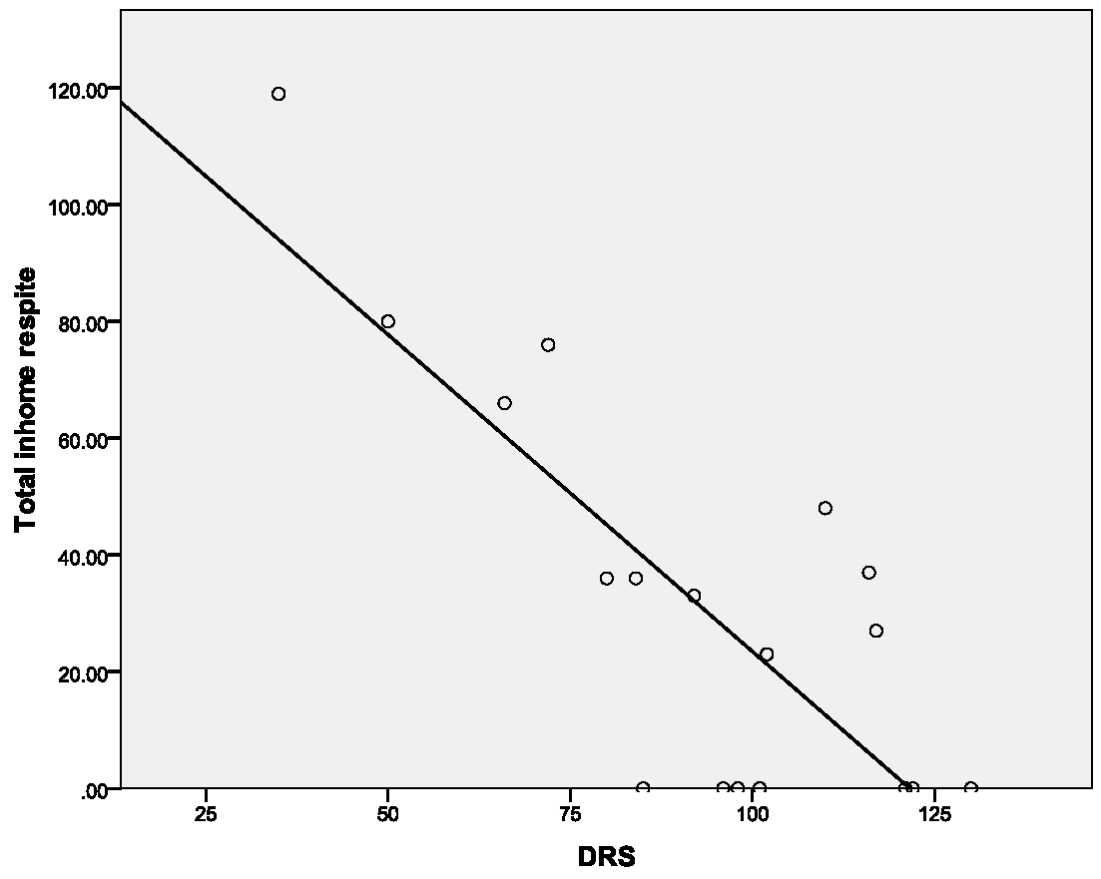
**Regression of hours of in-home respite by DRS-2**. Regression of the total hours of in-home respite received by carers over a 12 week period by the Dementia Rating Scale - 2 of the care recipients where less IHR services were related to higher DRS-2 scores (better cognition).

The relationships between measures of *normative *and *expressed *need were limited. Counter-intuitively, there was no relationship between dementia severity indicators (BADLS; DRS-2; dementia related problems via carer checklist) and out-of-home respite or practical care. In contrast, a negative correlation (Spearman's rho = 0.646, p = 0.01) did exist between DSR-2 scores and in-home respite hours, meaning that in-home respite service use increases as cognition deteriorates in the care recipient, (See Figure 1). The total BADLS scores also had a moderate correlation with in-home respite hours (Spearman's rho = 0.574, p = 0.01), suggesting that both deterioration in cognition and function are related to the need for in-home respite. Severity of cognitive impairment, behavioural and psychological symptoms, and impairment to everyday living in the care recipients bore no relationship to the amounts of practical care and OHR used by carers.

### Carers' felt needs

More than half of carers said they would like more help from services than they were currently receiving (*felt *need). Importantly, there was a correlation between *felt *need and carers' objective measures of stress (*normative *need). The carer rated item - 'I need more help from services than I am given' was positively related to carers' GHQ-30 (Spearmans rho = 0.625, p = 0.01), as was carers' 'I need more information than I am given' (Spearmans rho = 0.557, p = 0.05), and the carer rated stress related to these unmet service needs (Spearmans rho = 0.503, p = 0.05) and information needs (Spearmans rho = 0.634, p = 0.01). These were the only significant correlations between carers' subjectively and objectively rated burden and *felt *service need. This relationship suggests that carer-rated *felt *service needs are a useful indicator of carer psychological stress, while carer subjective burden is not. Significantly, carer *felt *need did not correlate with service use (*expressed *need), implying a high level of unmet service need.

We found that some carer characteristics were related to service use, namely that carers other than spouses were more likely to receive practical help (tau _b _= 0.535, p = 0.01), and that carers aged over 75 years were more likely to be using out-of-home respite (tau _b _= 0.33, p = 0.05).

### The suitability of services supplied

The qualitative data provided insights into why carers had unmet service needs and why dementia severity and carer stress were not directly linked to out-of-home respite or practical assistance received. A wide range of concerns led to resistance to, or refusal of, services, even for some objectively 'stressed' carers (i.e high GHQ-30). While the benefits of respite (time-out, opportunity to complete other chores etc.) were generally acknowledged, the cost/benefit balance for out-of-home respite was sometimes too high, because of the 'effort' needed to get the care recipient to an out-of-home respite facility, the emotional burden of guilt or worry, or the financial cost. The effort involved in helping to prepare care recipients with more advanced dementia for visiting an external service could be too great, for reasons ranging from functional incapacity, resistance, agitation, or unreliable transport services. Some care recipients with milder dementia did not like to attend out-of-home respite, and carers, did not like to (or could not) force them, as in the following example:

*"I've talked to her about the [OHR] and she just won't have a bar of it" *[(2) Int 2].

Carers felt 'caught' and 'trapped' when care recipients were reluctant to leave the home, as most care recipients could not be left alone. As a consequence, our sample of carers needed in-home respite as dementia severity increased, in order to address the basic requirements of their lives and households. One interviewee caring for a person with a DRS-2 score of 35 (i.e severe dementia) reported that:

*"With [IHR] I can leave the house to go out and do the things that I am interested in" *[(13) Int 2].

Another carer felt that in-home respite was necessary because:

*"You just go out, pay your bills, (then) you've got to get back again because you can't leave them on their own, they're not safe to be on their own" *[(8) Int 3].

In instances of in-home respite, carers also felt reassured that their 'time-out' had not been obtained at the cost of distressing the care recipient by exposure to new environments and unfamiliar circumstances.

The interviews also suggested reasons why practical help was the least used service in this sample. Most of the carers were female spouses, and for many (but not all) of this group, caring fell within their normative expectations of the spousal role. The increased 'work' within the home (for example, extra cooking and washing) was accepted as an extension of the regular duties that this role implied. For carers such as this one, offers of practical help were deemed unnecessary and inappropriate:

*"All I ever wanted was someone to be here in the house so he was safe, to feed him. I never expected or wanted anyone to come in and do my housework or any of those types of thing." *[(13) Int 1].

This quote illustrates the difference between the carer's felt need and a normative assessment of need, demonstrating that the carer is quite clear about the type of service she would like to receive and would accept.

The interviews supported documented evidence that care-giving is stressful, but also showed that carers' stress may originate from events that are unrelated to the cognitive or functional status of care recipients. The carer sample proffered examples of other events that had generated stress in their lives, such as a carer diagnosed with a life-threatening illness or with a medical history of mental illness, the death of a pet, and grief for the loss of the partnership that the care recipient had once provided. These diverse life situations and expectations highlight problems inherent in basing carers service needs on measures of carer burden or care recipient disease status, and explain why *normative *measures of need may fail to identify carers experiencing significant stress.

The interviews also indicated that the process of finding out about existing services and about their own eligibility for those services could be onerous for carers. Some carers felt that assessments were time consuming and achieved no satisfying result, either because particular services were not what the carer wanted or because the care recipient was not deemed eligible. The following quote is from a carer whose care-recipient had only moderate cognitive impairment, but whose GHQ-30 score was 13:

*"Well there's been five actual assessments and three interviews... over the last four months...then there was the day care lady came and assessed her and that was fruitless" *[(3) int 3].

This quote also demonstrates how many resources can be applied to *normative *need assessments (five home visits), without necessarily benefiting stressed carers. Overall, the interview data highlighted the complexity of interactions between the care-giving situation and service use, and the limitations of relying on professional assessments.

## **Discussion**

A key implication arising from our data is that *felt *needs expressed by carers of people with dementia are an important indicator of service need. The extensive list of possible causes of burden in carers' lives extends beyond the fact of caring for someone with dementia. Within the context of particularly challenging life circumstances, even modest care recipient demands may 'tip the scales' for carers and cause excessive stress. This means that relying on assessments of the status of care recipients with dementia, and on carer burden, may inadvertently exclude those for whom the basis of their need for services falls outside existing measures. While large data sets indicate that key factors such as cognition and ADL functions increase the probability of carers needing services [19], unknown and unpredictable contextual conditions also need to be taken into account. The significant correlation between our carers' mental health status and their stated need for more services suggests that *felt *need should be given greater priority over normative need in assessing service needs for carers of people with dementia.

Our interview data shed light on the limited relationship between *normative *measures of need and the *expressed *need of service use in the case of dementia carers. Insufficiently acknowledged interactions between carer life circumstances and identity issues, and the disease status of care recipients may mean that measures of *normative *need capture only a limited range of causes of carer stress and service needs. Measuring stress clinically with a more direct tool such as the GHQ-30 objectively measures carer stress, since professionals are not trying to 'work backwards' by assessing possible causes of stress, in the manner of many burden measures. Given the complexity of carers' life situations, and the benefits that carers provide to the health care system in supporting people with dementia, it may be that offers of services should be based primarily on carers *felt *needs. This suggestion is supported by previous studies finding that unmet service needs have complex causes [20], and that direct questions of unmet need were better predictors of impending admission to care homes or death of care recipients than other assessments [19].

The correlation between DSR-2 and BADLS and in-home respite suggests that the cognitive and functional aspects of care recipient deterioration are linked to in-home respite need. The reported social isolation of spouse carers [13] is an explanation supported by the study's qualitative data, complementing Mahoney's [[32]: 221] finding that carer burden emerges from the constant "vigilance" that is required of the carer, in order to protect and care for the person with dementia.

Assisted by the provision of only modest service hours, carers who participated in this study were able to support care recipients to remain at home until they reached moderate to severe stages of dementia. Carers such as these make an important contribution to the health care system and save health and social services significant costs thereby benefiting the public purse. In line with previous research [9,10] however, we found that caring for a person with dementia brings social, emotional, physical and financial costs to the carers themselves, with our participants identified as more stressed than their non-care-giving peers according to the GHQ-30. This stress highlights the importance of addressing carers unmet service needs, if policy makers wish them to maintain care in the home for extended periods. Our results suggest that carers' stated (*felt*) service needs should be considered a 'red flag' by service providers.

What of those who refuse services? These findings suggest carers could be refusing services because particular services are not suitable, or because the carer does not identify his or her stress as directly linked to the care recipient. In these cases, services may need to offer more flexible options, a conclusion also reached by other researchers [14]. These carers are also likely to benefit from approaches that involve them in decisions about services and provide choice. Having choice and being able to participate in service decisions are two factors desired by health service users [33] and found to delay entry to care homes for care recipients [34], but these options were missing in the experiences of our participants. The complexity of circumstances surrounding each carer indicates that they must be allowed to play a more pivotal role in need assessments, and that services need to offer service choices that are flexible and responsive. In order to have choice, carers firstly require information about service availability. This is a confusing area even for experienced health professionals [35], which, as a crucial first stage in service provision, must be rendered less daunting for all stakeholders as a matter of priority. Consistent with other studies [29,36,37] we found that carers may be unaware of available services and would be likely to benefit from greater knowledge of available forms of assistance. Overall, it is likely that the provision of more comprehensive information, engagement in service needs assessments, and allowance for choice and service flexibility constitute the first steps to be taken in decreasing service refusal in situations of need.

The small convenience sample used in this study is a limitation that raises the risk of Type II errors and prevents generalisability. The participants comprising our sample were already linked into and using some services, as could be expected by the dementia severity of their care recipients, and did not include isolated carers, or carers of persons with early stage dementia. Our sample also consisted mostly of female spouses, and this clearly influenced their belief systems about the appropriateness of using services. However the mixed method design we utilised provides a different strength through triangulation of quantitative with qualitative data, in which interlinked contextual information informed the interpretation of measurement results.

## **Conclusions**

Overall, this exploratory study suggests that *normative *need is not as useful as *felt *need when considering the health service needs of carers of people with dementia. A focus on measuring the care recipient's disease status and carer's burden overlooks the influences that broad life circumstances have on carer's service needs. Attempts to index the carer's service needs via objective cataloguing of functional and cognitive impairments may therefore be inadequate Our data suggest that *felt *need is a suitable indicator of carer unmet needs, because it is significantly related to carers' mental health status. *Felt *need may therefore be an appropriate 'red flag' of carer burnout, even in the absence of obvious "flags" raised by behavioural, functional and cognitive decline. In view of the current client-centred focus of health services, and the acknowledged burgeoning of numbers of people with dementia, our study contributes to the growing knowledge of how services can better support carers. Health services and professionals may need to reorient their approach away from traditional normative need assessments toward more participatory *felt *need. The small sample of carers and care recipients used in this mixed-methods study means that a larger investigation is required to assess the relevance of our findings to the general population of carers of people with dementia. However the similarity of our findings to larger data sets suggests that such a wider investigation needs to focus on *felt *carer need and service access, rather than on assessment.

## Competing interests

None declared. This study was funded by the JO and JR Wicking Trust (ANZ Charitable Services). The funders played no role in the study, data analysis or writing of the article. All researchers had complete independence from the funders in undertaking the study.

## Authors' contributions

CS and SA analysed the data and wrote the paper. TC, AR, JV and PT designed and supervised the study. All authors read and approved the final manuscript.

## Pre-publication history

The pre-publication history for this paper can be accessed here:

http://www.biomedcentral.com/1472-6963/10/122/prepub
